# Effect of flaxseed oil on biochemical parameters, hormonal indexes and stereological changes in ovariectomized rats

**DOI:** 10.1002/vms3.372

**Published:** 2020-10-25

**Authors:** Romina Tanideh, Shirin Delavari, Omid Farshad, Cambyz Irajie, Mohammad Javad Yavari Barhaghtalab, Farhad Koohpeyma, Omid Koohi‐Hosseinabadi, Akram Jamshidzadeh, Nader Tanideh, Aida Iraji

**Affiliations:** ^1^ Student Research Committee Shiraz University of Medical Sciences Shiraz Iran; ^2^ Pharmaceutical Sciences Research Center Shiraz University of Medical Sciences Shiraz Iran; ^3^ Department of Medical Biotechnology, School of Advanced Medical Sciences and Technologies Shiraz University of Medical Sciences Shiraz Iran; ^4^ Department of General Surgery Shahid Beheshti Hospital, Yasuj University of Medical Sciences Yasuj Iran; ^5^ Endocrinology and Metabolism Research Center Shiraz University of Medical Sciences Shiraz Iran; ^6^ Laparoscopy Research Center Shiraz University of Medical Sciences Shiraz Iran; ^7^ Central Research Laboratory Shiraz University of Medical Sciences Shiraz Iran; ^8^ Stem Cells Technology Research Center Shiraz University of Medical Sciences Shiraz Iran; ^9^ Department of Pharmacology, School of Medicine Shiraz University of Medical Sciences Shiraz Iran; ^10^ Medicinal and Natural Products Chemistry Research Center Shiraz University of Medical Sciences Shiraz Iran

**Keywords:** flaxseed oil, medicinal plants, ovariectomy, phytoestrogens, polyunsaturated fatty acids

## Abstract

The ovariectomized rat is a widely used preclinical model for studying postmenopausal and its complications. In this study, the therapeutic effect of flaxseed oil on the ovariectomized adult rats was investigated. Our results showed that biochemical parameters including calcium, oestrogen and progesterone levels increase 8 weeks after ovariectomy in rats. Also, the amount of alkaline phosphatase decreased significantly after 8 weeks compared with the OVX rat. The healing potential of flaxseed oil was proven by successfully recovering the affected tissue and preventing the unpleasant symptoms of ovariectomized rats. The biological effects of flaxseed oil may be due to high amounts of fatty acids, phytoestrogens and an array of antioxidants. The results suggest that flaxseed oil can mimic the action of oestrogen and can be a potential treatment for hormone replacement therapy (HRT).

## Graphical abstract

1. The potential use of flaxseed oil as the natural source for hormone replacement therapy on the ovariectomized rat model was investigated.
2. The antioxidant properties and biochemical parameters including alkaline phosphatase, calcium, estrogen, and progesterone levels were determined in ovariectomy rats.

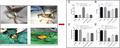

## 1 INTRODUCTION

Ovariectomy (OVX) regarded as one of the most common surgical procedures in the world with approximately one third of women all around the world (Laughlin‐Tommaso et al., 2014). These signs and symptoms of ovariectomy include fever, redness, swelling, vomiting, urinating difficulty, chronic abdominal pain, shortness of breath or chest pain, mood swings as well as deficiency of ovarian hormones (Coleman et al., 2011; Kurita et al., 2016). Ovarian hormones play a pivotal role in maintaining the integrity and function of female genital tissue structure, and shown to enhance genital sensation, maintain blood flow, rugae of the vaginal wall as well as vaginal lubrication (Traish et al., 2018; Tzur et al., 2016). OVX alongside its diverse effects on alterations of lipid metabolism and loss of oestrogen signalling also results in an increased risk of heart disease, atherosclerosis, obesity, inflammation and osteoporosis (Leung, 2012). As a result, developing new candidate or improving diet by modifying the amount and type of fat ingested, which in turn improves lipid profile without side effects, have raised the prospect of new intervention with a much lower cost than drugs.

Substantial, surveys in humans and animals suggested that dietary phytoestrogens have protective effects against menopausal symptoms and a variety of disorders, including cardiovascular disease, cancer, hyperlipidemia, osteoporosis and various forms of chronic renal disease (Bhathena & Velasquez, 2002).

Phytoestrogens are found in numerous fruits, seeds and whole grains and are categorized into three classes including the isoflavones, lignans and coumestans. Phytoestrogens with a similar chemical structure to endogenous oestrogen can bind to oestrogen receptors and affect the glucose, lipid metabolism and increase the absorption of calcium (Sirotkin & Harrath, 2014) which in return result in improving memory, neuronal survival, reproductive behaviour, emotion and sexual differentiation (Raheja et al., 2018). Some studies also reported the pain modulation role of phytoestrogens (Valsecchi et al., 2008; Wong et al., 2017).

Flaxseed (linseed, *Linum usitatissimum*, Linaceae) is the seed from the flax plant (*Linum usitatissimum L*.) and is widely used as a powerful natural product in many parts of the world due to the potential health benefits associated with some of its biologically active components. Flaxseed oil is naturally rich sources of polyunsaturated fatty acids (PUFA), especially omega‐3 and omega‐6 fatty acids, lignans (secoisolariciresinol diglycoside‐SDG), soluble antioxidants and insoluble fibres (Goyal et al., 2014; Ivanov et al., 2011; Singh et al., 2011; Thompson et al., 1996; Touré & Xueming, 2010). Flaxseed oil is a safe and healthy supplement that can boost intake of omega‐3 fatty acids from seafood and lower cholesterol as well as chances of developing cardiovascular diseases (Bloedon & Szapary, 2004), diabetes, risk of cancer especially the breast and prostate gland (Dabrosin et al., 2002), inflammation (Hallund et al., 2008) and osteoporosis (El‐Saeed et al., 2018). Various studies demonstrated no evidence of acute toxicity of flaxseed oil after 90 days of oral, dermal and inhalation consumption, and no LD_50_ was reported (Baker & Grant, 2018; Marambe et al., 2017).

The aim of this study was to investigate the efficacy of the flaxseed oil on the lipid profiles, biochemical, hormonal indexes in serum and uterine stereological in ovariectomized (OVX) rats. Flaxseed oil is an exceptionally safe natural product and a rich source of PUFA specifically in ω‐3 fatty acids, phytoestrogenic lignans and an array of antioxidants. We hypothesized that flaxseed oil administration as a nutritious meal would regulate the biochemical and hormonal pathway and have a positive impact on the ovariectomized rats as a model of human menopause.

## 2 MATERIALS AND METHODS

### 2.1 Raw material and oil extraction

Flaxseed (*linum usitatissimum L*.) was obtained from the Agriculture Research Center, Shiraz, Iran. Flaxseeds were cleaned and kept in paper bags at room temperature until further analysis. Flaxseed oil was obtained by filter press flaxseeds in which the seeds were pressed in a disc press and subsequent filtrated. The oil was stored at −20°C for later use (Tanideh, et al., 2020).

### 2.2 Determination of the antioxidant activity of flaxseed oil

The method consisted of spectrophotometric measurement of the intensity of the colour change in a solution containing 2,2‐diphenyl‐1‐picrylhydrazyl (DPPH). The test sample solutions (20 μL) containing different concentrations of the oil in MeOH were mixed with a methanolic solution of DPPH (180 µM). This mixture was incubated at room temperature for 30 min, and finally, absorbance was measured at 517 nm with a spectrophotometer (Ardakani Movaghati et al., 2019; Koohi‐Hosseinabadi et al., 2017). Each experiment was repeated 3 to 5 times and all the data were presented as means ± standard errors (SE). Methanol was regarded as a blank for the microplate reader. Quercetin was used as the reference antioxidant with the half‐maximal inhibitory concentration (IC_50_) of 9.1 ± 0.42 µM. The results of the DPPH assay were expressed as IC_50_ values by the Curve Expert software (for Windows, version 1.34) (Ostovar et al., 2020).

### 2.3 Animals

All the procedures were in agreement with the guidelines of the Committee in Research Ethics of the Shiraz University of Medical Sciences (No. 2225b125). Fifty adult female Sprague‐Dawley rats, aged 6 months, weighing between 200 ± 20 g were purchased from the laboratory animal's centre of Shiraz University of Medical Sciences. The rats were maintained under standard housing laboratory conditions, the relative humidity of 60 ± 5%, the temperature of 23 ± 2°C and 12‐hr light/dark cycles and were fed with a standard pellet diet and water ad libitum. After 1 week of adaptation to the diet and the new environment, the animals were screened. Then, the rats were randomly divided into five groups each containing 10 animals:

- Group 1, control (received 2.50 mg/kg body wt almond oil orally);
- Group 2, sham operated (received 2.50 mg/kg body wt almond oil orally);
- Group 3, OVX as a negative control (received 2.50 mg/kg body wt almond oil orally);
- Groups 4, OVX + Estradiol rats as a positive control (received 1 mg/Kg estradiol and 2.50 mg/kg body wt and almond oil orally);
- Groups 5, OVX + Linum rats as a treated group (received 2.50 mg/kg body wt flaxseed oil and 2.50 mg/Kg almond oil orally).

### 2.4 Ovariectomy

Bilateral ovariectomy (OVX) and sham operation were undergone anaesthesia with ketamine 10% (100 mg/kg, Alfasan, Netherlands) and xylazine 2% (10 mg/kg, Alfasan, Netherlands). After ligation of the uterine horn through a midline longitudinal incision, both ovaries were surgically removed in all the groups, except for the control and sham groups. The sham‐operated rats had their ventral incision, but the manipulation of ovaries was performed without excising them.

### 2.5 Biochemical tests

The biochemical assessment was performed after 8 weeks of treatment by flaxseed oil. Blood samples were collected in chilled non‐heparinized tubes, centrifuged at 3,500 rpm at 4°C for 20 min (Tanideh, et al., 2020). The separated sera were separated and evaluated for biochemical markers, including calcium, and alkaline phosphatase (ALP) through the calorimetric methods (Dabbaghmanesh et al., 2017). Oestrogen and progesterone levels were also quantified by enzyme‐linked immunosorbent assay (ELISA) analytical biochemical technique. Analyses of all samples, standards and controls were performed in duplicate.

### 2.6 Stereological measurements

The Cavalieri method was applied to estimate the total volume of the uterine horn. To estimate the volume, the uterine horn was divided into 8–12 parallel sections with a specified distance (*t*). After tissue processing, the sections were placed in paraffin blocks. Serial 5‐μm‐thick sections were taken using a microtome and stained with Hematoxylin and Eosin (H&E) (Merck company, Germany) method.

**FIGURE 1:**
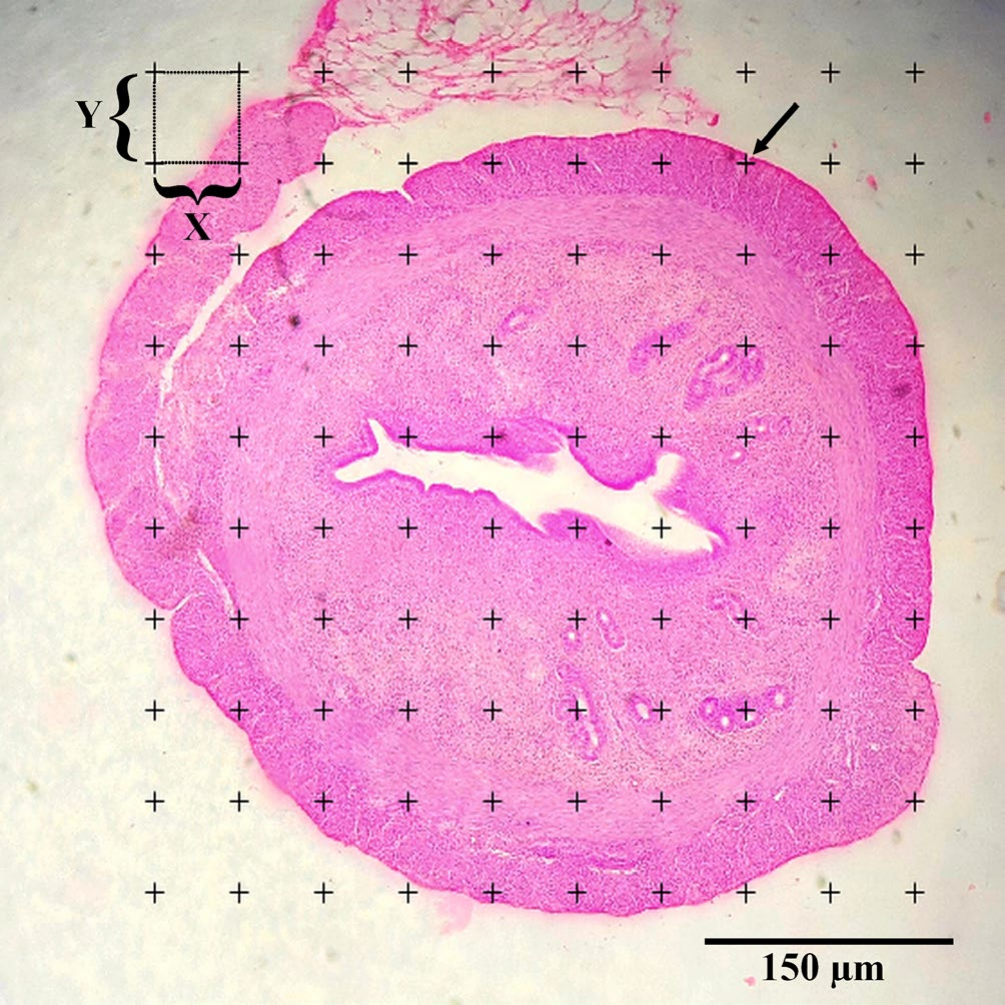
Point counting method was used to estimate the volume of different layers of the uterine horn, lumen volume, and volume density of uterine gland. The arrow indicates the right upper quadrant of each cross that was considered to be a point. H&E staining with magnification at ×40

A point grid was used to calculate the slice area, which was completely randomized to the cutting surface. First, calculate the area around each point (a(p)=ΔX×ΔY). In the grid, then estimate the area of each slice by multiplying the total number of the points on the grid that hitting the slices $\left(\sum_{i=1}^{n}p\right)$. Then, the total volume is calculated by multiplying the aggregate areas in slices thickness (Figure 1). Briefly, the total volume of the uterine horn was estimation using the following formulas:

$$V_{\text{total uterine horn}}=\sum_{i=1}^{n}p\times a(p)\times t$$

Volume density of the targeted structure (here, Perimetrium, Myometrium, Endometrium, Lumen and Uterine gland) was estimated on 5‐µm‐thickness sections through the point‐counting method and using Delesse's formula (Noorafshan et al., 2013)(Figure 1):

$$Vv\left(\text{structure}\right)=\sum_{i=1}^{n}p(\text{structure})/\sum_{i=1}^{n}\left(\text{reference}\right)$$

“$\sum_{i=1}^{n}p(\text{structure})$” was the number of the test points falling on the targeted structure (here, Perimetrium, Myometrium, Endometrium, Lumen and Uterine gland) and “$\sum_{i=1}^{n}p(\text{reference})$” was the total points hitting the uterine horn sections. The following formula was used to estimate the absolute targeted structure volume (Noorafshan et al., 2015):

V(structure)=V(total uterine horn)×Vv(structure).

#### 2.6.1 Estimation of the different layer's thickness of the uterine horn

**FIGURE 2:**
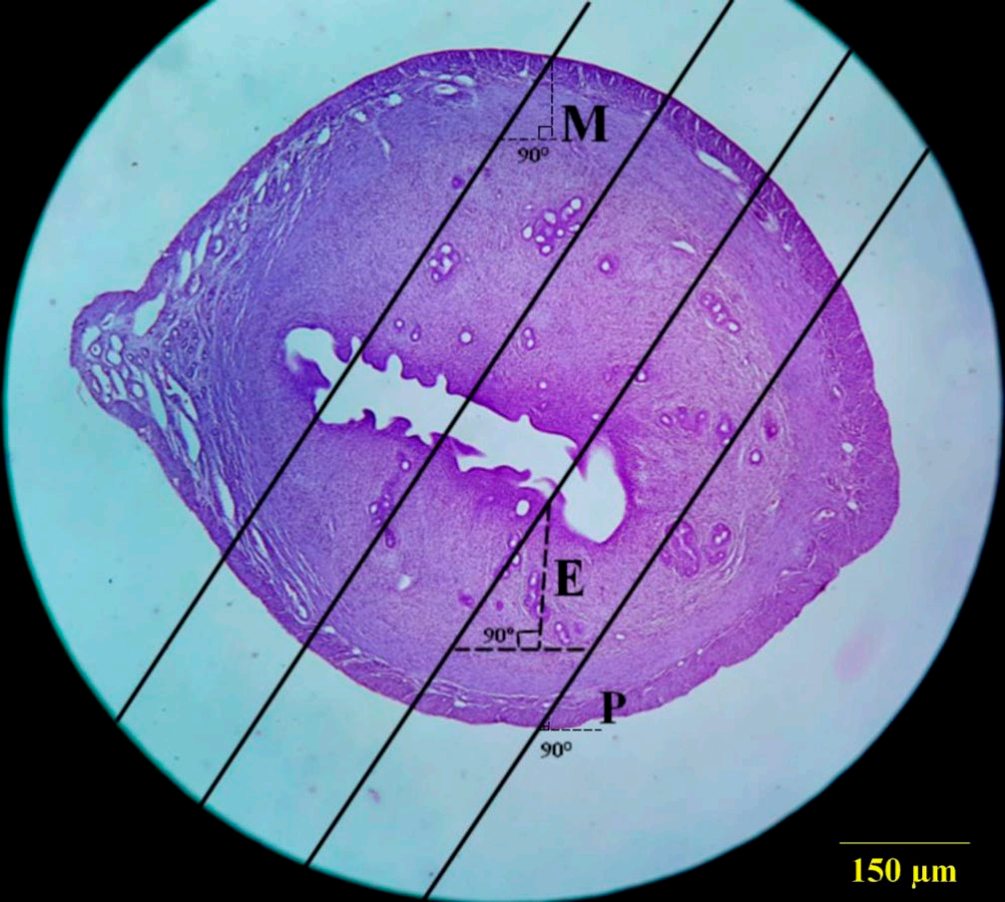
The orthogonal intercept method for measurement of the different layers of the uterine horn thickness P) Perimetrium thickness, M) Myometrium thickness, E) Endometrial thickness. H&E staining with magnification at ×40

To evaluation, the mean thickness of the different layer's thickness of the uterine horn, an average of 8–12 sections from 5‐µm‐thick sections were randomly selected and studied using the Nikon microscope (e200, Japanese) with a ×100 magnification. To determine measurement sites, the specific line grid (4 parallel lines) was randomly superimposed on the sampled fields (Figure 2). To calculate the mean thickness of the perimetrium, myometrium and endometrium, the orthogonal intercept method was used. The specific line grid (four parallel lines) was randomly superimposed on the sampled fields. The length of a perpendicular line extended from the inner layer to the outer layer of the uterine horn at each intercept of the line of the grid with the outer layer considered as the orthogonal intercept. An average of 100–200 measurements was estimated and the harmonic mean thickness was calculated using the following formula.

$$\text{zona pellucida mean thicknes}=\frac{8}{3\pi }\times \text{number of measurements}/\left(\frac{1}{oi_{1}}+\frac{1}{oi_{2}}+\frac{1}{oi_{3}}+\ldots \right)$$

Orthogonal intercepts: number of measurements.

### 2.7 Statistical analysis

The SPSS statistical software (v. 18) was used to carry out statistical analysis. First, the normal distribution of the data was assessed. Then, the data were analysed by the Mann–Whitney U test. The results were presented as mean ± standard deviation, and the significance level was set as *p* ≤ .05.

## 3 RESULTS

### 3.1 Antioxidant activity

Free radical scavenging activity was defined as the amount of antioxidant necessary to decrease and inactivate the initial DPPH radical to 50% in 30 min (IC_50_ ± *SD*). The DPPH radical scavenging activity expressed as the IC_50_ of the oil was 26.32 ± 4.52 µg/ml and quercetin as positive control demonstrated IC_50_ of 9.1 ± 0.42 µM.

### 3.2 Biochemical parameters

#### 3.2.1 Alkaline phosphatase levels

**FIGURE 3:**
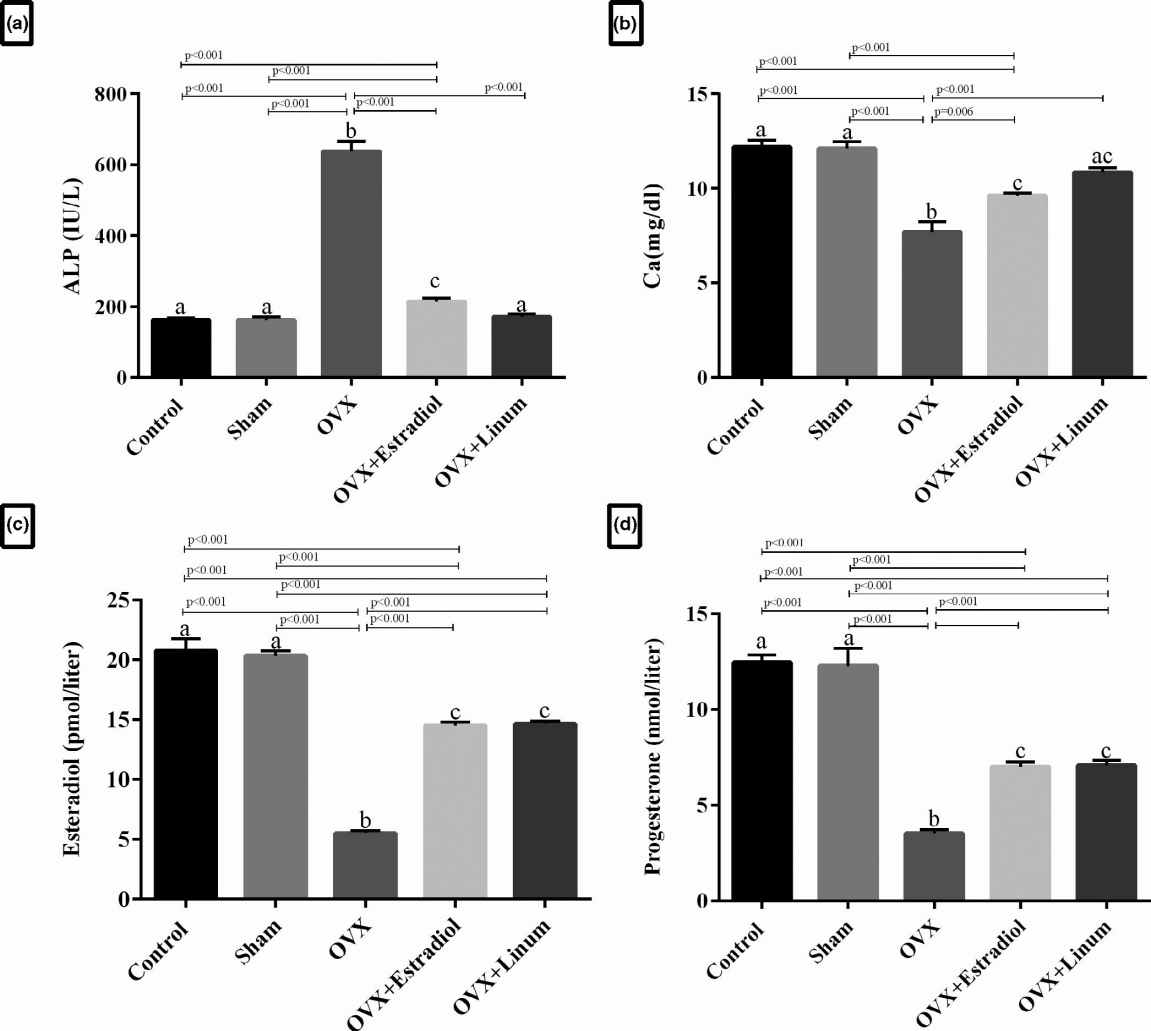
Evaluation of biochemical parameters in experimental groups. (a) ALP level on experimental groups, (b) Serum calcium level on experimental groups, (c) Estradiol levels on experimental groups, (d) Progesterone levels on experimental groups Data are express as Mean ± *SEM*. a,b,c: According to the posthoc Tukey test which used for intergroup comparisons, groups with same superscripted letters were not significantly different at *α* = 0.05(*p* ≥ .05). However, dissimilar letters indicate a significant difference (*p* < .05). ac letters above the error bars of the histogram show no significant difference among mentioned group and a and c groups

As predicted in Figure 3, A. plasma ALP level significantly increased in the OVX rats compared with the sham‐operated and control groups (*p* < .001). A significant decrease in the ALP levels was observed in the flaxseed oil‐treated rats and the estradiol‐treated rats compared with OVX group (*p* ≤ .001). Interestingly, the flaxseed oil‐treated group (OVX + Linum rats group) brought even more decrease in the ALP levels compared with OVX + Estradiol group as standard, although the change was not significant.

#### 3.2.2 Calcium levels

Serum calcium levels in the experimental groups have been shown in Figure 3, B. As depicted, ovariectomy caused a significant decrease in the calcium level of the OVX group compared with the sham and control (*p* < .001). Remarkably, the administration of flaxseed oil increased calcium levels during the treatment with a *p*‐value <.001 compared with the OVX group. No significant difference was observed between the flaxseed oil‐treated rats and the estradiol‐treated rats.

#### 3.2.3 Oestrogen and progesterone levels

The levels of both oestrogen (*p* < .001) and progesterone (*p* < .001) were significantly decreased in the OVX group compared with the control and sham groups (Figure 3C and D). However, the level of oestrogen and progesterone in the treatment groups with OVX‐estradiol and flaxseed oil was significantly increased in comparison with the OVX group (*p* < .001).

### 3.3 Stereological study

#### 3.3.1 Effect of flaxseed oil on uterus weight and volume

**FIGURE 4:**
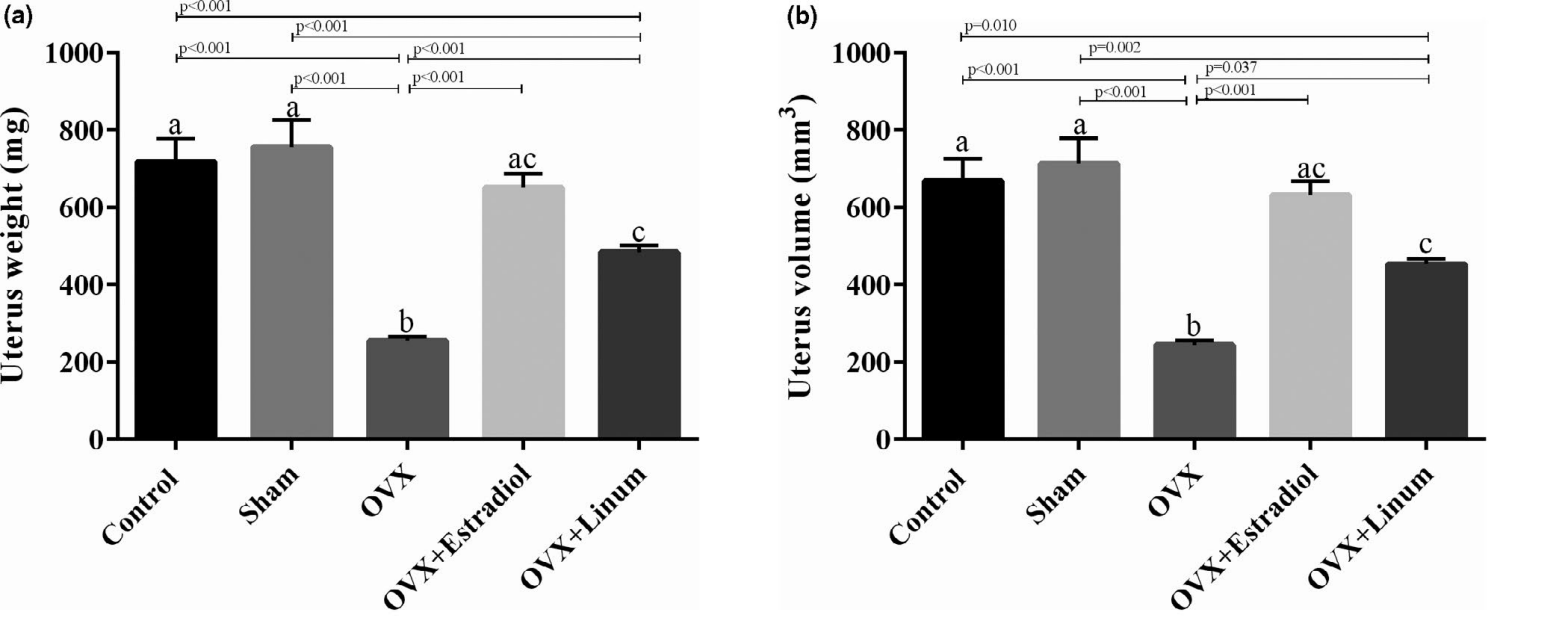
Effect of flaxseeds oil on uterus weight (a) and volume (b). According to the posthoc Tukey test which used for intergroup comparisons, groups with same superscripted letters were not significantly different at *α *= 0.05 (*p* ≥ .05). However, dissimilar letters indicate a significant difference (*p* < .05). ac letters above the error bars of the histogram show no significant difference among mentioned group and a and c groups

As depicted in Figure 4. A, an increase in the uterus weight was evident in the OVX + estradiol and OVX + Linum groups in comparison with the OVX (*p* < .001). Also, statistical analysis did not show any significant difference between the uterus weight of positive control and the treated group. Similarly, the uterus volume of OVX + Linum group also demonstrated an increase compared with the OVX group (*p* = .037).

#### 3.3.2 Effect of flaxseed oil on perimeter ovarian thickness and volume

**FIGURE 5:**
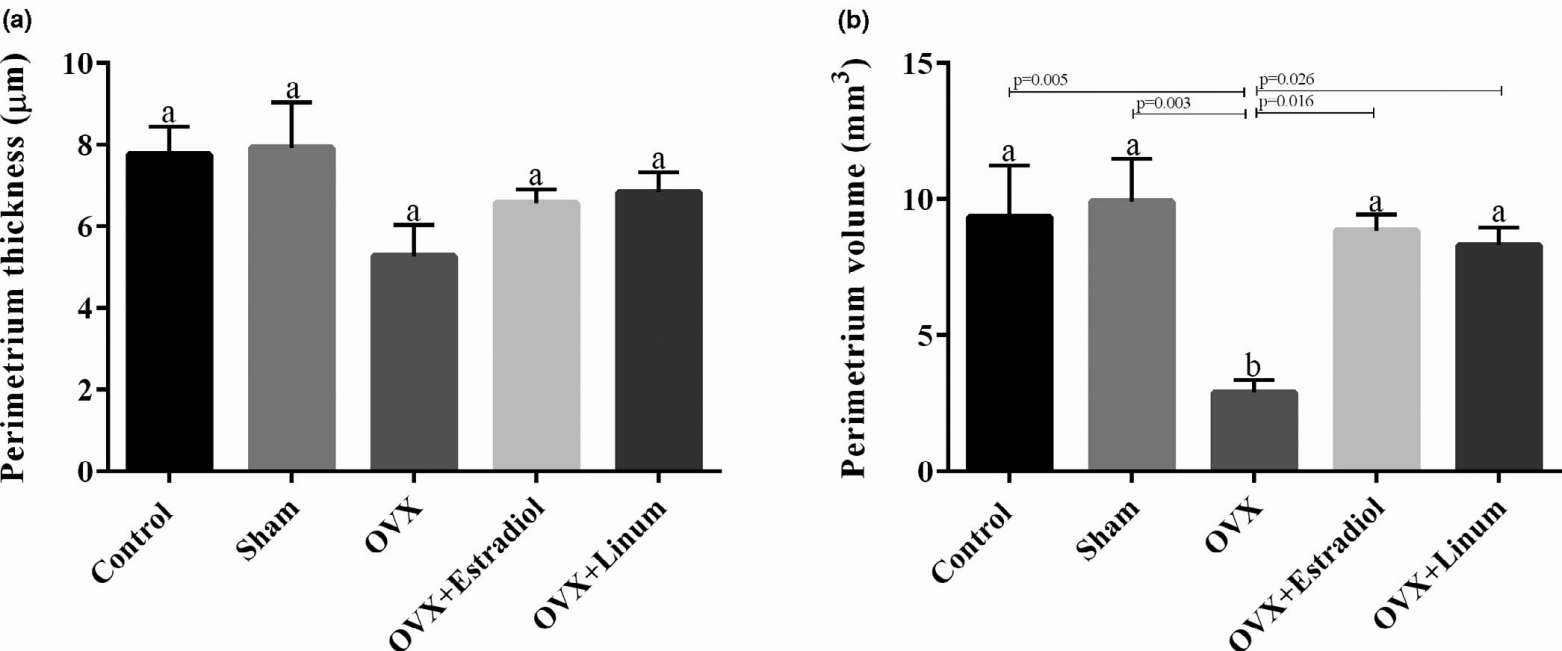
Effect of treatment on perimeter ovarian thickness (a) and volume (b). According to the posthoc Tukey test which used for intergroup comparisons, groups with same superscripted letters were not significantly different at *α *= 0.05(*p* ≥ .05). However, dissimilar letters indicate a significant difference (*p* < .05)

Similar, but less clear effects were observed on the perimeter ovarian thickness compared with endometrial. The perimetrium thickness was reduced by ovariectomy; however, the intake of flaxseed reversed it to normal conditions (Figure 5A). In the case of perimeter ovarian volume (Figure 5B), it can be seen that the treatment of rats with flaxseed oil and estradiol enhanced the volume with a *P*‐value of .026 and .16, respectively, compared with OVX group. No significant difference was observed between the flaxseeds oil‐treated rats and the estradiol‐treated rats.

#### 3.3.3 Effect of flaxseed oil on endometrial thickness and volume

**FIGURE 6:**
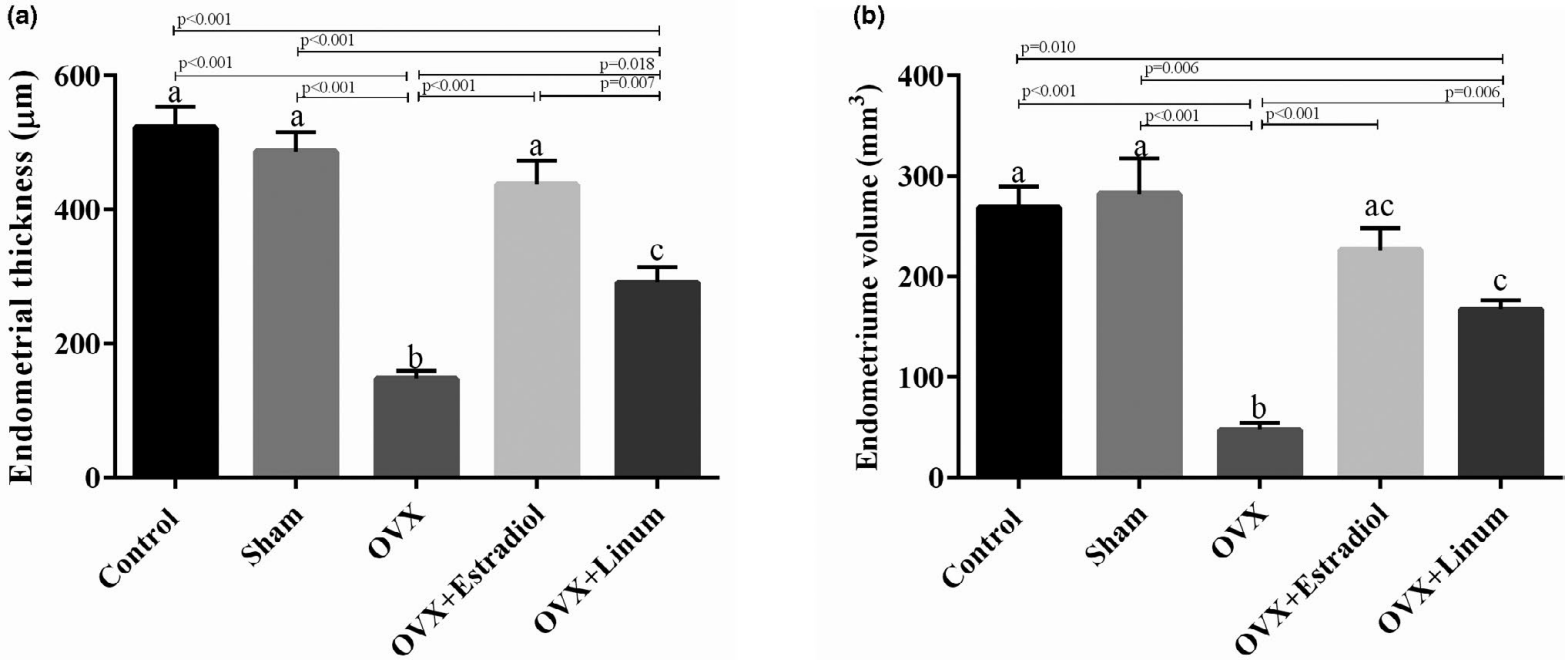
Effect of treatment on endometrial thickness (a) and volume (b). According to the posthoc Tukey test which used for intergroup comparisons, groups with same superscripted letters were not significantly different at *α *= 0.05(*p* ≥ .05). However, dissimilar letters indicate a significant difference (*p* < .05). ac letters above the error bars of the histogram show no significant difference among mentioned group and a and c groups

Data in Figure 6. show that ovariectomy decreased the endometrial thickness and volume significantly concerning the control and sham‐operated groups (*p* < .001). The administration of flaxseed oil appears to improve these parameters.

#### 3.3.4 Effect of flaxseed oil on myometer ovarian thickness and volume

**FIGURE 7:**
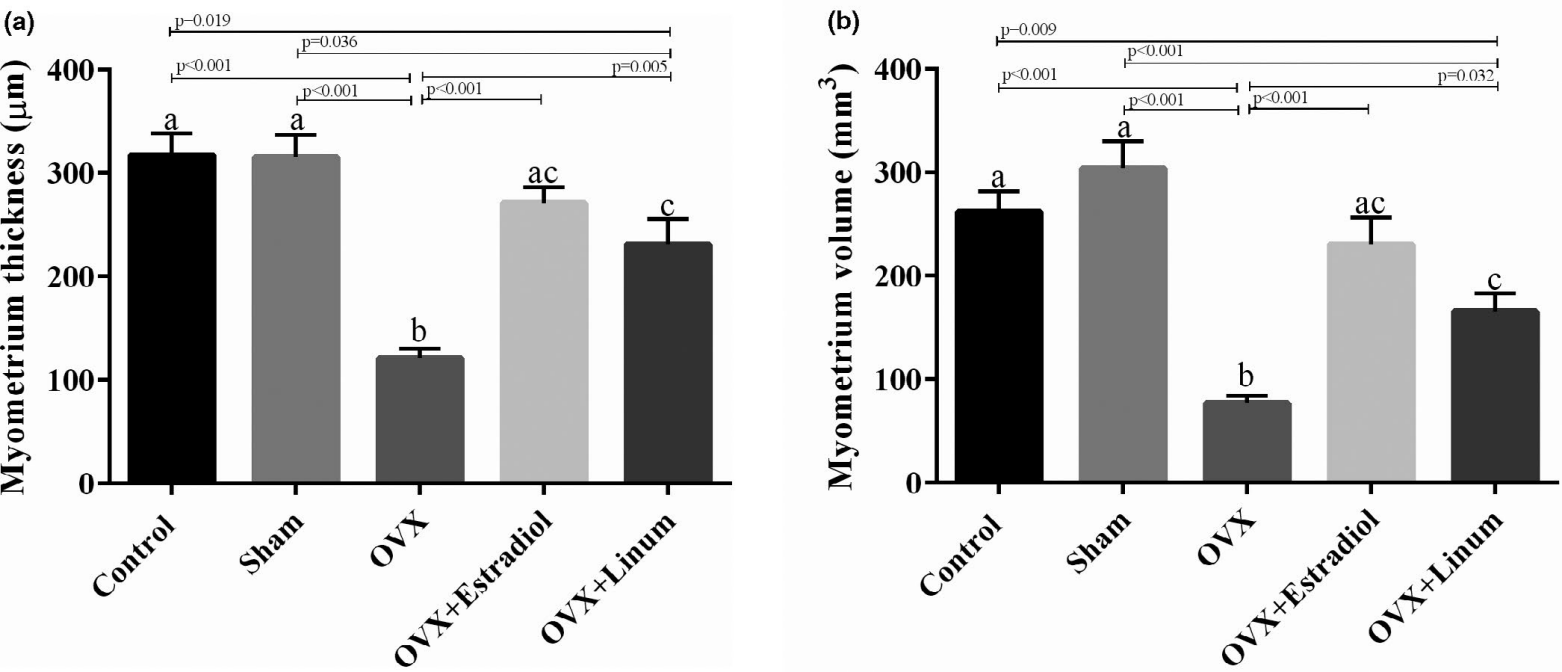
Effect of treatment on myometer ovarian thickness (a) and volume (b). According to the posthoc Tukey test which used for intergroup comparisons, groups with same superscripted letters were not significantly different at *α *= 0.05(*p* ≥ .05). However, dissimilar letters indicate a significant difference (*p* < .05). ac letters above the error bars of the histogram show no significant difference among mentioned group and a and c groups

As might be expected, ovariectomy decreased the myometer thickness (Figure 7A) and volume significantly with *P*‐value <.001 (Figure 7B). Flaxseed oil administration improved the mentioned parameters in such a way that an acceptable increase compared with the OVX group was seen (*p* = .005 for myometer thickness and *p* = .032 for myometer volume compared with the OVX group).

#### 3.3.5 Effect of flaxseed oil on vessel ovarian diameter

**FIGURE 8:**
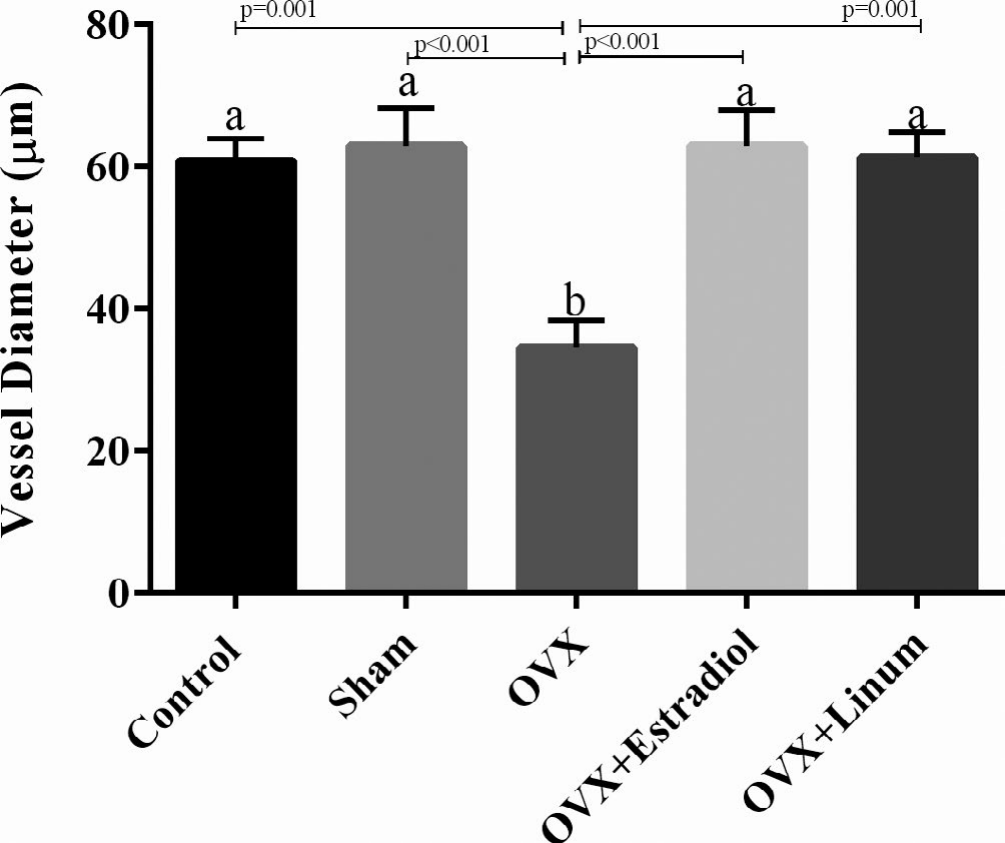
Effects of Effect of treatment on vessel ovarian diameter. According to the posthoc Tukey test which used for intergroup comparisons, groups with same superscripted letters were not significantly different at *α *= 0.05(*p* ≥ .05). However, dissimilar letters indicate a significant difference (*p* < .05)

Based on the data presented in Figure 8, ovariectomy decreased the vessel ovarian diameter significantly compared with the control group (*p* = .001). Administration of flaxseed oil meaningfully increased the vessel ovarian diameter compared with the OVX group (*p* = .001) and returned these parameters to around normal conditions without significant differences compared with the control group.

#### 3.3.6 Effect of flaxseed oil on lumen volume

**FIGURE 9:**
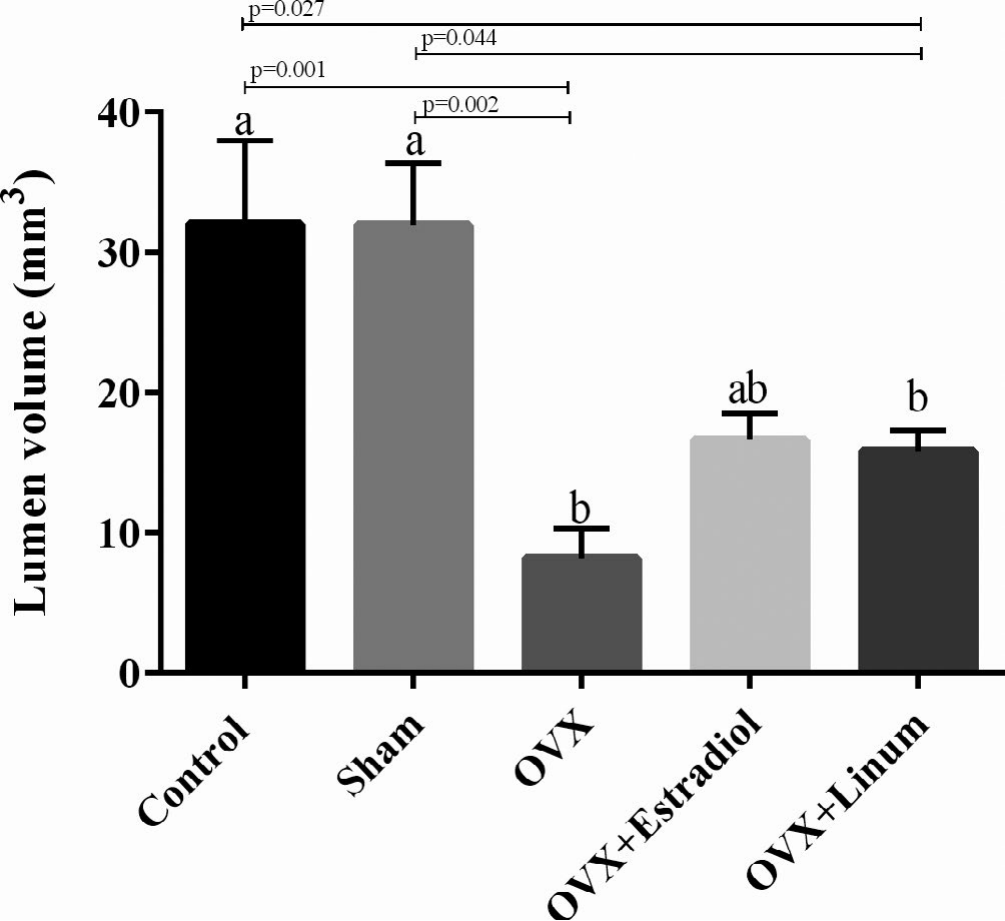
Effects of Effect of treatment on lumen volume). According to the posthoc Tukey test which used for intergroup comparisons, groups with same superscripted letters were not significantly different at *α *= 0.05(*p* ≥ .05). However, dissimilar letters indicate a significant difference (*p* < .05).ab letters above the error bars of the histogram show no significant difference among the mentioned group and a and b groups

It is well known that lumen volume reduced by ovariectomy. Administration of flaxseed oil tended to increase the lumen volume, but these changes were not statistically significant (Figure 9.).

### 3.4 Histopathological study

**FIGURE 10:**
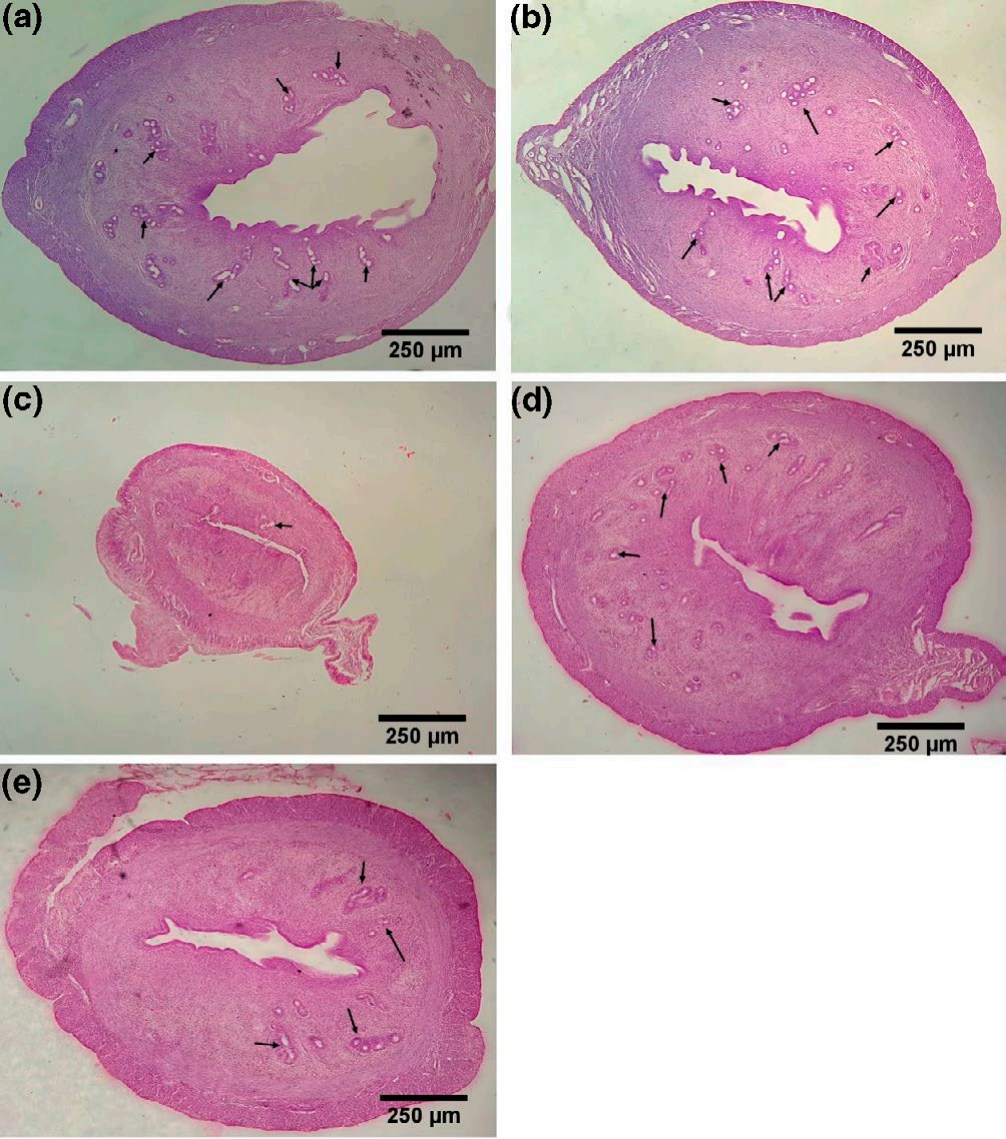
Histopathological study of uterus tissue and the effect of estradiol and Flaxseeds oil in uterus tissues in ovariectomized rats. A: Control group, B: sham group, C: OVX group, D: OVX + estradiol group, E: OVX + Linum group. (Fig A–E: H & E staining with magnification at ×4). The results showed severe atrophy and a decrease in the volume, thickness of the layers and glands of the uterine tissue in the OVX group (Figure C) compared to the other groups

As depicted in Figure 10, in the treated groups, increasing of the volume uterus, the thickness of the layers and glands (arrow mark) are well visible. There were no significant changes in the sham group compared with the control group.

## 4 DISCUSSION

The purpose of this study was to reduce the complications of menopause and post‐ovarian problems in women. Ovariectomy is well known to decreased oestrogen hormones, calcium metabolism, bone mineral content, endometrial and lumen weight and volume. These diverse effects could also result in bone fracture tissue called osteoporosis (Lello et al., 2018; Sattar, 2016; Taylor et al., 2019). The Food and Drug Administration (FDA) approved OVX rat model to study menopause, postmenopausal osteoporosis and the possible natural and synthetic therapeutic agents (Johnston & Ward, 2015).

The main issue is that oestrogen and progesterone changes due to ovariectomy and alter biochemical factors including alkaline phosphate and calcium level (Ghazvini et al., 2016; Shen et al., 2017). Oestrogen is lipophilic steroid hormone that binds to nuclear oestrogen receptors (ERα and ERβ), regulates gene expression within DNA, activates gene transcription and stimulates steroidal hormone (González‐Bengtsson et al., 2016). Additionally, oestrogens can activate the cell surface G‐protein coupled Oestrogen Receptor (GPER) which promotes cyclic AMP (cAMP) and lesser intracellular Ca^2+^ (Nilsson et al., 2011; Sharma et al., 2018). However, the exact contributions of oestrogen signalling through ERα, ERβ or GPER related to lipid and lipoprotein metabolism are not well defined (Palmisano et al., 2018). In return, the loss of oestrogens in adult animals is associated with body weight gain and the development of obesity (Chen et al., 2009). Moreover, deficiency of oestrogen reduces thiol antioxidant defences in bone cells and the expression of TNFα, which prompt apoptosis and causes loss of bone (Jagger et al., 2005; Lean et al., 2003). Also due to the reduction in ovarian hormones, uterine structure and ovarian thickness changes and as a result endometrial atrophy happened. Another problem in ovariectomized women is oxidant/antioxidant imbalance that can exacerbate their well‐being. There is increasing evidence suggesting the destructive role of free radicals oxidative damage on cells and bone (Muthusami et al., 2005). Menopause happens due to the loss of ovarian follicular function, decreases follicle‐stimulating hormone and oestrogen levels. These may result in bone turnover, urogenital and menstrual cycle disorders, vasomotor changes and unpleasant symptoms such as irritability, arthralgias, sleep disturbances and mood swings (Das, 2002). However, the exact mechanism of bone metabolism by oestrogens is not clear. The protective effect of oestrogens on bone mass maintenance may be the result of nonnuclear initiated signalling of the ERα (Almeida et al., 2017). H_2_O_2_ generated in the mitochondria of osteoclasts is required for the loss of cortical bone mass caused by oestrogen or androgen deficiency (Ucer et al., 2015, 2017). As a result consumption of high amounts of antioxidants can markedly improve functions in ovariectomized cases.

For centuries flaxseed, has been cultivated considering its potential health benefits and medicinal purposes. In recent decades, flaxseed oil has been studied for many potential uses including kidney disorders, anti‐inflammation, bacterial and fungal infection, diabetes, cardiovascular diseases and cerebrovascular stroke (Goyal et al., 2014; Madhusudhan, 2009).

Moneim et al showed that consumption of 1,000 mg/kg flaxseed oil in rats not only depicted no toxicity but also limit the renal cytotoxicity (Abdel Moneim et al., 2011). The other studies also confirm the potential protective influence of flaxseed oil against renal toxicity through alteration biochemical and histopathological factors (Shaikh Omar, 2018). These data confirmed the low toxicity and therapeutic potency of the mentioned oil.

The high interest in consumption of flaxseed oil as a medicinal and nutritional product is mainly due to its high PUFA content (especially omega‐3 and omega‐6 groups), lignans, high‐quality proteins, fibres and carbohydrates. Although the composition of flaxseed can vary with genetics, growing environment, seed processing and method of analysis (Bernacchia et al., 2014; Rubilar et al., 2010), PUFAs are a family of lipids identified by the position of the last double bond in their structure. Several studies confirmed that flaxseed oil is a rich source of essential PUFA such as linoleic acid and α‐linolenic acid which regulate prostaglandin synthesis and, hence, enhance the wound‐healing process. Besides omega‐3, characterized as the most known PUFA, improve the development of the nervous system, reducing cardiovascular disease and cancer. Omega‐3 is also known to lessen the severity and minimize symptoms of chronic inflammatory and inflammatory bowel disease. However, direct evidence of potentially beneficial effects of dietary PUFA on human osteoporosis is still lacking. Omega‐3 fatty acids consumption reduces plasma triglycerides (TG) levels, and small dense LP while increasing large buoyant LDL and HDL‐C, may result in an improved lipid profile (Pirillo & Catapano, 2013). The role of omega‐3 fatty acid supplementation in dyslipidemia treatment is also shown in other studies (Maki et al., 2009; Saito et al., 2008; Tanaka et al., 2008).

The primary investigation depicted that in ovariectomized female rats, the bone weight of femur and tibia decreased significantly. Interestingly, the PUFA‐enriched diet prevented the loss of bone weight and strength caused by oestrogen deficiency, as would occur in women during post‐ovary problems (Sakaguchi et al., 1994). In another research project, oil containing a high amount of PUFA acts as antagonistic to arachidonic acid with prostanoid action, which conserves and also stimulate bone mineral content in adult especially during oestrogen deficiency (Watkins et al., 2003).

Different mechanism explains the synergic effect of oestrogen and PUFAs. First, the beneficial actions of oestrogen depend on PUFA, specifically, PUFA increases the sensitivity of receptors towards oestrogen and can modulate the binding of hormones (even at very low concentration) to their nuclear receptors and produced appropriate and enough response. As a result, when the levels of PUFAs decrease, the binding of oestrogen to its receptors will not be optimum, leading to suboptimal responses (Das, 2002). Second, oestrogen and PUFAs mostly interact with each other in such a way that the action and regulation of one depend on other molecules. The correlation between them could be enhanced during the overproduction of NO which plays an important role in atherosclerosis, osteoporosis, neurodegenerative conditions and memory (Das, 2002; Van der Kraan et al., 2000). PUFAs may be key to the actions of oestrogen due to altering the expression of receptors on the cells. Hence, a combination of PUFAs and oestrogen may be necessary to optimize their benefit and response (Nordoy et al., 1998; Nordøy et al., 2000). Third, PUFAs are involved in the anti‐osteoporotic action of oestrogen *via* mediating some of the actions of TGF‐β (Newman, 1990).

PUFAs also well defined as anti‐inflammatory agents through inhibiting the production of proinflammatory cytokines IL‐1, IL‐2 and TNF‐α (Das, 2000). PUFA modifies the actions of some of the growth factors that influence immune response and decrease free radical generation (Das, 2001; Mohan & Das, 2000, 2001). Studies have evaluated the biological mechanism of PUFA on bone loss. Recent research highlighted that long‐term intake of n‐3 PUFA increasing leptin and IGF‐1 levels and improved the mechanical properties of cortical bone (Rahman et al., 2009). The other study suggested that PUFA was shown to down‐regulate osteoclastogenic factors and reduce bone loss in ovariectomized mice (Kang et al., 2004). These data are in accordance with our results which demonstrated the high biochemical capacity of the mentioned oil.

The other compounds in flaxseed are Lingnan phytoestrogens. Phytoestrogens are herbal steroids that have an oestrogen‐like structure that binds to the ER‐α and ER‐β and produces a response without negative effects of synthetic oestrogens (Harris et al., 2005; Strauss et al., 1998) (Strauss et al., 1998). Studies on phytoestrogens about bone health in postmenopausal women and prostate health in men have shown promising results (Dalais et al., 1998; van de Poll, 2004; Marini et al., 2007).

The DPPH method is an easy and simple method to estimate the ability of antioxidants to scavenge free radicals. Evidence of antioxidant potential of flax was reported in lots of studied (Rajesha et al., 2006; Touré & Xueming, 2010). Recently, Lucas et al. showed that flaxseed is beneficial in reducing plasma cholesterol and plaque formation induced by ovarian hormone deficiency. Flaxseed reduced the fatty streak area and the incidence of lesions to levels (Lucas et al., 2004). Saarinen et al. reported consumption of flaxseed reduced the tumour growth in ovariectomized mice and long‐term consumption of phytoestrogen‐rich diet stimulates the growth of oestrogen (Saarinen et al., 2006). The protective effect of flaxseed oil in the ovariectomized rat was also reported by Boulbaroud (Boulbaroud et al., 2008).

The mechanisms by which flaxseed improves biochemical, stereological parameters and the mechanical property in the lumen are not clear. The high potency of flaxseed may be due to high PUFAs and oestrogen‐like compounds alongside its antioxidant capacity. The current study demonstrated the significant increase in calcium and alkaline phosphate level due to the consumption of flaxseed which is close to the control and sham group. Also, oil had a therapeutic impact on ovarian thickness and volume. Besides, flaxseed oil antioxidants could be another factor that lowers the side of OVX. The data in this study showed this oil can be used as a complementary and low‐cost treatment to reduce the dose and side effects of estradiol during menopause after ovarian problems. A closer look at published data confirms a close and accurate connection between our results and published data.

The main finding of this study was that flaxseed oil treatment prevented the impairment and disorders induced by ovariectomy. However, further studies are required to elucidate the mechanism of action and its potential use for the treatment of postmenopausal osteoporosis. The different dosage forms of flaxseed oil including tablets, syrups and the transdermal patch can be developed to evaluate the effectiveness, biocompatibility and bioavailability of the mentioned oil. Also, MRI analysis can be performed as the diagnostic and prognostic techniques to determine the structural changes related to the uterus, ovaries and fallopian tubes before and after drug administration. In the next step, the clinical trial study can be conducted to ensure that medical intervention and treatment are safe and effective. These findings including pharmacological and biochemical results could be useful to change lifestyle strategies such as diet among postmenopausal and OVX women.

## 5 CONCLUSIONS

The findings of this study provided new insights into the effects of flaxseed oil on human health as hormone‐replacement therapy (HRT) attenuate symptoms and also prevent OVX and menopause. The potential use of flaxseed oil as natural sources may be due to high amounts of PUFA and antioxidants. The present study proposes an ideal natural agent that could restore the balance between biochemical and hormonal parameters and reduced harmful effects on target tissues.

## CONFLICT OF INTEREST

The authors of this manuscript declare no conflict of interests.

## AUTHOR CONTRIBUTION

Romina Tanideh: Formal analysis; Investigation; Visualization; Writing‐original draft. Shirin Delavari: Data curation; Formal analysis; Methodology; Resources. Omid Farshad: Data curation; Formal analysis. Cambyz Irajie: Project administration; Software; Supervision; Writing‐review & editing. Mohammad Javad Yavari Barhaghtalab: Formal analysis; Methodology; Validation; Writing‐original draft; Writing‐review & editing. Farhad Koohpeyma: Formal analysis; Visualization. Omid Koohi‐Hossenabadi: Data curation; Formal analysis; Methodology. Akram Jamshidzadeh: Conceptualization; Project administration. Nader Tanideh: Conceptualization; Investigation; Project administration; Supervision; Writing‐review & editing. Aida Iraji: Conceptualization; Formal analysis; Investigation; Methodology; Supervision; Writing‐review & editing.
